# Cardiac Mechanics and Ventricular Twist by Three-Dimensional Strain Analysis in Relation to B-Type Natriuretic Peptide as a Clinical Prognosticator for Heart Failure Patients

**DOI:** 10.1371/journal.pone.0115260

**Published:** 2014-12-29

**Authors:** Sheng-Nan Chang, Yau-Huei Lai, Chih-Hsuan Yen, Chia-Ti Tsai, Jou-Wei Lin, Bernard E. Bulwer, Ta-Chuan Hung, Charles Jia-Yin Hou, Jen-Yuan Kuo, Chung-Lieh Hung, Juey-Jen Hwang, Hung-I Yeh

**Affiliations:** 1 National Taiwan University College of Medicine, Graduate Institute of Clinical Medicine, Taipei City, Taiwan; 2 Division of Cardiology, Department of Internal Medicine, National Taiwan University Hospital Yun-Lin Branch, Dou-Liu City, Taiwan; 3 Division of Cardiology, Department of Internal Medicine, Mackay Memorial Hospital, Taipei, Taiwan; 4 Division of Cardiology, Department of Internal Medicine, National Taiwan University Hospital, Taipei, Taiwan; 5 Noninvasive Cardiovascular Research, Cardiovascular Division, Brigham and Women's Hospital, Boston, Massachusetts, 02115, United States of America; 6 Mackay Medical College, New Taipei City, Taiwan; 7 The Institute of Health Policy and Management, College of Public Health, National Taiwan University, Taipei City, Taiwan; Temple University, United States of America

## Abstract

**Background:**

Three dimensional (3D) echocardiography-derived measurements of myocardial deformation and twist have recently advanced as novel clinical tools. However, with the exception of left ventricular ejection fraction and mass quantifications in hypertension and heart failure populations, the prognostic value of such imaging techniques remains largely unexplored.

**Methods:**

We studied 200 subjects (mean age: 60.2±16 years, 54% female, female n = 107) with known hypertension (n = 51), diastolic heart failure (n = 61), or systolic heart failure (n = 30), recruited from heart failure outpatient clinics. Fifty-eight healthy volunteers were used as a control group. All participants underwent 3D-based myocardial deformation and twist analysis (Artida, Toshiba Medical Systems, Tokyo, Japan). We further investigated associations between these measures and brain natriuretic peptide levels and clinical outcomes.

**Results:**

The global 3D strain measurements of the healthy, hypertension, diastolic heart failure, and systolic heart failure groups were 28.03%, 24.43%, 19.70%, and 11.95%, respectively (all *p*<0.001). Global twist measurements were estimated to be 9.49°, 9.77°, 8.32°, and 4.56°, respectively. We observed significant differences regarding 3D-derived longitudinal, radial, and global 3D strains between the different disease categories (*p*<0.05), even when age, gender, BMI and heart rate were matched. In addition, 3D-derived longitudinal, circumferential, and 3D strains were all highly correlated with brain natriuretic peptide levels (*p*<0.001). At a mean 567.7 days follow-up (25^th^–75^th^ IQR: 197–909 days), poorer 3D-derived longitudinal, radial, and global 3D strain measurements remained independently associated with a higher risk of cardiovascular related death or hospitalization due to heart failure, after adjusting for age, gender, and left ventricular ejection fraction (all *p*<0.05).

**Conclusions:**

3D-based strain analysis may be a feasible and useful diagnostic tool for discriminating the extent of myocardial dysfunction. Furthermore, it is able to provide a prognostic value beyond traditional echocardiographic parameters in terms of ejection fraction.

## Introduction

Heart failure (HF) is a growing epidemic and this problem is largely due to an increasing elderly population [1]. Despite modern medical advancements, mortality and clinical outcomes from both systolic and diastolic heart failure (DHF) remain poor [2]. Along with age, hypertension (HTN) and several other clinical risk factors for myocardial damage and subsequent HF development have been identified [3], [4], [5], [6].

New imaging techniques that are able to improve our understanding of cardiac mechanics and assist in the early identification of myocardial dysfunction are rapidly evolving, and it is hoped that they will improve the health care delivery and therapeutic strategies for HF populations [7], [8], [9]. Currently, myocardial contractile function remains the key index in evaluating the severity and prognosis of HF [10], [11], [12], [13], [14]. However, echocardiography-derived linear and 2-dimensional (2D) measures of the left ventricular ejection fraction (LVEF) can be heavily load dependent, with scant information provided regarding regional functionality during the early stages of myocardial decline (e.g., for those subjects with DHF) [15], [16].

2D speckle tracking imaging (2D-STI) is an angle-independent technique for measuring myocardial deformations (longitudinal, radial, and circumferential) and twist. Previous studies have demonstrated that 2D-STI accurately detects early changes to left ventricle (LV) contractile function before LVEF deterioration, and that it can provide new insights into the understanding of global and regional systolic myocardial mechanics [15], [16], [17]. Despite its advantages over conventional LVEF measurements, 2D-STI is limited by foreshortened LV views and assumptions involving spatial or geometric parameters [17]. In addition, the significance of these parameters on clinical outcomes remains largely unknown.

With the advantages of frame-by-frame analysis, and the full volume acquisition of whole LV cavities, real-time 3 dimensional (3D) echocardiography has the potential to circumvent the aforementioned limitations of 2D-STI [18]. In this regard, 3D echocardiography-based myocardial deformation and twist behavior may provide a more accurate and comprehensive assessment of cardiac mechanics to avoid out-of-plane motion, and may provide additional information regarding various clinical scenarios [19].

Previous studies have shown that HTN is a risk factor for myocardial damage and subsequent HF, especially DHF. However, the exact mechanistic alterations that occur during this process remain largely unknown [3]. We therefore utilized 3D echocardiography to characterize and explore the alterations to cardiac mechanics and twist behavior during the transition from a diagnosis of HTN to HF development. Furthermore, we investigated the correlations of such measures to clinical outcomes, and examined their clinical prognostic values beyond traditional echo parameters.

## Materials and Methods

### Study subjects

This was a single center-based retrospective study, comprising consecutive patients (baseline cohorts) that had been referred for transthoracic echocardiography at a tertiary medical center in Taipei, Taiwan (1^st^ cohort: 2 months enrollment period from June 1, 2009 to Aug 1, 2009. 2^nd^ cohort: 1 month enrollment period from April 1, 2014 to May 1, 2014. See S1 Fig. for a study flow chart). All patients with sufficient quality echocardiography 2D images were enrolled. The entire study cohort comprised subjects with diagnosed HTN and HF, with either preserved (DHF) or reduced (systolic heart failure, SHF) LV systolic function. This study was approved by the ethics/IRBs committee on human research of the Medical Research Department, Mackay Memorial Hospital, Taipei, Taiwan. (Board Number: 11MMHIS144). According to the regulations of the ethics/IRBs committee, collections of non-invasive materials for routine practice and data prepared retrospectively for future publications required verbal inform consent if necessary. The data collected from the participants was de-identified during data processing. According to the regulations of ethics/IRBs committee on human research at our institution, retrospective data analyses did not require further documentation. Inclusion criteria were those subjects with HTN and HF. This information was obtained either from medical histories or from noting the regular control medications. For the HTN group, subjects with a known HTN diagnosis but without any clinical evidence or history of HF were enrolled [20]. Subjects with a known clinical HF hospitalization history based on Framingham criteria, were categorized by global LVEF into either the SHF or DHF group [20]. Fifty-eight healthy volunteers served as a control group in this study. Baseline clinical information, biochemical data, and medical histories were all collected using an electronic chart review system.

All patients underwent both 2D and 3D echocardiography examinations. Patients were excluded from the study on the basis of poor acoustic windows, atrial fibrillation or other significant arrhythmias, previous cardiac pacemaker implantation, recent or acute coronary events (within 6 months), significant valvular heart diseases (moderate degree or above), overt renal failure (serum creatinine level ≥3 mg/dl), malignancy with expected survival <1 year, scheduled cardiac surgery in the near future, and probable difficulties involving follow-up.

### Echocardiographic studies based on 2D and Doppler measurements

2D images were obtained using standard parasternal and apical views with frame rates of 80–100 frames/s, with a GE Vivid 7 ultrasound system (GE Vingmed Ultrasound, Hortan, Norway) equipped with a 2.5–3.5 MHz broadband transducer. M-mode measurements of inter-ventricular septum (IVS), relative wall thickness (RWT), and LV mass were performed. The LV mass index (g/m^2^) was defined as the ratio of the LV mass to body surface area (BSA) [21], [22]. Left atrium (LA) and LV volumes (both end-diastolic and end-systolic) were further quantified using the 2D biplane Simpson's method. Assessments of LV diastolic function were performed using pulsed wave Doppler parameters, with transmitral inflow velocities measured at the tip of the mitral leaflets. Early (E) and late diastolic (A) filling velocities, mitral inflow E/A ratios, deceleration times (DT), and isovolumic relaxation times (IVRT) were all determined. High frame-rate spectral tissue Doppler imaging (TDI) analysis was used to determine both septal and lateral mitral annulus velocities by averaging the peak systolic (S′) and early diastolic (E′) values for three consecutive beats (both septal and lateral mitral annulus). The pulmonary capillary wedge pressure (PCWP) was estimated by dividing the early (E) transmitral Doppler velocity by E′ (E/E′).

### Featured 3D tracking algorithm

3D echocardiography was performed using an Artida ultrasound system (Artida, Toshiba Medical Systems, Tokyo, Japan) with full volume electrocardiogram (ECG)-gated 3D datasets (S2 Fig.) acquired from the apical positions by a matrix phased-array 2.5 MHz transducer (PST–25SX). The most suitable location for 3D full volume dataset acquisition on the chest wall was determined using prior 2D images acquisition site from the same subject. During one breath-holding, the depth and sector width were adjusted to minimize the values for optimal spatial and temporal resolution of the entire LV within the pyramidal volume.

In the tissue harmonic mode, 3–4 wide-angled acquisitions were made consisting of four wedge-shaped sub-volumes acquired over four consecutive cardiac cycles. These were automatically integrated into a wide-angle (70°×70°) pyramidal dataset with the highest frame rate achievable (20–26 Hz). The data were stored and transferred for off-line analysis. From three consecutive acquisitions, the most optimal image dataset (as determined by an experienced cardiologist) was chosen for subsequent analysis. While analyzing the images, the epicardial and endocardial borders at the end-diastole for the 2- and 4 chamber views, were semi-automatically traced using the Artida software. This method has previously been validated using sonomicrometry [23], and was conducted with minimal manual adjustment by the same experienced investigator who was blinded to the clinical information.

3D echocardiographic parameters, specifically the left ventricular end diastolic volumes (LVEDV), left ventricular end systolic volumes (LVESV), LVEF, global 3D strains, 3D longitudinal strains (LS), 3D radial strains (RS), 3D circumferential strains (CS), and LV twists were then analyzed automatically by the Artida software (Toshiba Medical Systems Co.). The automatic analyses of strain and twist are referred to in S2, S3, and S4 Figs.

The values of 3D-based myocardial strain/deformation components were calculated as percentage changes by the following formula: strain  =  (L1 - L0/L0) ×100 [%], where L1 is the segment length at end-systole, and where L0 represents the segment length at end-diastole. Based on this formula, all 3D-derived myocardial deformation parameters, including LS, RS, and CS, were calculated from different directions or planes of myocardial motion. The global 3D strain was derived by tracking two adjacent points, one on the endocardium and the other on the epicardium, based on a real 3D algorithm from all tracking points distributed on the entire LV myocardium throughout the cardiac cycle, regardless of planes of motion [18]. Based on this data, the results of global 3D strains are expressed as an average percentage value from all these vector motions throughout the whole LV in the same subject.

For 3D LV twist calculations, the data was expressed as a degree value representing the short axis apex-to-base gradient of rotation. The measurement of various myocardial deformations and twist using 2D speckle-tracking techniques has been described previously [24].

The intra- and inter-observer variability (ICCs) of 3D strain measurements for 18 subjects randomly chosen from this study was 0.89 and 0.85, respectively. The reproducibility of the values obtained in this study was thus shown to be high.

### Biomarkers for brain natriuretic peptides and renal function

Venous blood samples were collected from all participants for biochemical analysis using a Biosite Triage Meter (San Diego, CA), on the same day of echocardiography. The estimated glomerular filtration rate (eGFR) was estimated by the Modification of Diet in Renal Disease (MDRD) equation [25].

### Statistical analysis

Continuous data were reported as the mean ± standard deviation (SD), and were compared using a nonparametric trend test (Wilcoxon rank-sum test) across the ordered disease category groups. Incidence data were expressed as a proportion, and were compared using either the chi-squared or Fisher's exact test, where appropriate. Differences in continuous data between groups was determined via a one-way analysis of variance (ANOVA) followed by the Bonferroni post hoc test for multiple paired comparisons. The statistical power was calculated in a pre-specified manner, with an alpha error of 5% capable of providing significant differences with a statistical power >90% for deformation data (including the SHF, DHF, and HTN groups) among at least 30 subjects, compared to the control population for each disease category. We further matched age, gender, BMI and heart rate between groups, in order to further disclose the effects of our target myocardial deformations or twist measurements after accounting for the potentially confounding effects of these key clinical variables.

Clinical outcomes were defined as HF hospitalization or cardiac death after the study date. The clinical follow-up period was pre-specified, with a follow-up interval of at least 2 years, using the echocardiography examination time as the index date. This was performed to assure that a sufficiently large number of subjects (>25%) would experience a clinical event. Receiver operating characteristic curve (ROC) analysis was used, with an optimal cut-off generated from the largest sensitivity and specificity summation for myocardial deformation strata. Discriminations of area under the ROC (AUROC and its 95% confidence interval: 95% CI) in clinical outcome predictions were performed by C-statistics. Multivariable Cox proportional hazard regression models were used to identify the independent role of myocardial deformation in predicting clinical outcomes, with hazard ratio (HR) and 95% CI reported. The likelihood ratio test was used for comparisons of the incremental values for different echo-derived parameter combination models. The original value of 3D LS and 3D CS acquired from 3D echocardiography, were presented in the same direction as 2D-derived LS and CS values (as negative). In order to present our data uniformly among all 3D-derived strain components and clinical correlates or outcomes, we calculated and expressed the “absolute values” of 3D LS and 3D CS (as positive) during our data analysis.

A two-tailed *p* value of <0.05 was considered statistically significant. Statistical analyses were performed with STATA software for Windows, version 9.0 (Stata Corporation, College Station, Texas) [26].

## Results

### Baseline characteristics of the study population

Of the 214 subjects initially enrolled, a total of 200 had complete baseline information and sufficient quality 3D images rendered for myocardial strain tracking, with baseline information according to different disease categories (control, HTN, DHF, and SHF) (Table 1).

**Table 1:** Baseline demographic characteristics of the study subjects.

Clinical Variables	Control (n = 58)	HTN (n = 51)	DHF (n = 61)	SHF (n = 30)	*p* value
Age, years	51.5±13.9	56.9±12.8	67.9±14.7* †	65.5±18.6*	<0.001
Gender, male	22 (38%)	30 (59%)	23 (38%)	18 (60%)	0.056
Body weight, kg	63.3±18	64.6±11.3	73.2±15* †	67.5±14.2	<0.001
BMI, kg/m^2^	23.6±3.5	24.9±3.5	28.6±4.4* †	26.1±5.2	<0.001
SBP, mmHg	127.4±18.6	139.2±22.5*	141.7±21*	137.4±26.5*	0.0021
DBP, mmHg	75±10.7	81.5±13.1*	78.7±13.4	77.1±14.5	0.0473
HR, 1/min	74.1±10.5	81.9±11.9*	77.4±13.1	84.9±15.5	0.0028
eGFR, ml/min/1.73 m^2^	94.9.7±23.3	88.2±27.2	74.6±28.3*	62.4±23.8* ^‡^	<0.001
BNP (log)	2.13±1.4	2.28±1.31	4.09±1.67* †	5.4±1.61* † ^‡^	<0.001
HTN, %	—	51 (100%)	46 (75%)	22 (73%)	<0.001
DM, %	—	16 (31%)	20 (33%)	10 (33%)	<0.001
Hyperlipidemia, %	—	17 (33%)	20 (33%)	11 (37%)	<0.001
CAD, %	—	7 (14%)	23 (38%)	17 (57%)	<0.001

*p<0.05 vs. control group;

† *p*<0.05 vs. HTN group; and ^‡^ *p*<0.05 vs. DHF group.

Abbreviations: BMI: body mass index, SBP: systolic blood pressure, DBP: diastolic blood pressure, HR: heart rate, eGFR: estimated glomerular filtration rate, BNP: brain natriuretic peptide, HTN: hypertension. DM: diabetes, CAD: coronary artery disease.

Subjects in the HTN, DHF, and SHF groups tended to be older, with higher SBP, and with poorer renal function (i.e., lower eGFR) as compared to the control group (all *p*<0.05). Compared to the other groups, subjects with DHF were older and had a higher BMI (both *p*<0.05). Additionally, patients with HF (both DHF and SHF) had significantly higher BNP levels than the other two groups (*p*<0.001), with the SHF group having the highest BNP (log 5.4±1.61). In addition, DM, hyperlipidemia, and coronary artery disease (CAD) were more prevalent in patients in the three diseased clinical categories.

### Comparisons and correlations between 2D- and 3D-derived myocardial strain measures and cardiac twist

**Figure 1:**
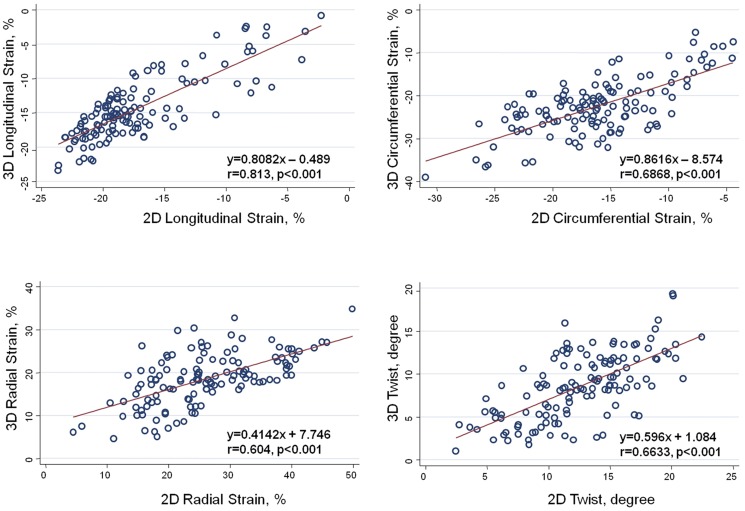
The comparison between various left ventricular deformations (strains) and twist using 2D and 3D echo methods. The correlations between 2D and 3D echo methods are also reported: LS (*r* = 0.813, *p*<0.001), CS (*r* = 0.687, *p*<0.001), RS (*r* = 0.604, *p*<0.001), and twist (*r* = 0.663, *p*<0.001).

Measurement comparisons of LV strain and torsion between the 2D and 3D echo methods are shown in Fig. 1. Using the correlation coefficient, the analysis revealed a close association between the 2D and 3D echo methods for 3D LS (*r* = 0.813, *p*<0.001), 3D CS (*r* = 0.687, *p*<0.001), 3D RS (*r* = 0.604, *p*<0.001), and 3D twist (*r* = 0.663, *p*<0.001).

### Comparisons of conventional echo-derived parameters and 3D-derived myocardial deformation and cardiac twist across the disease categories

**Figure 2:**
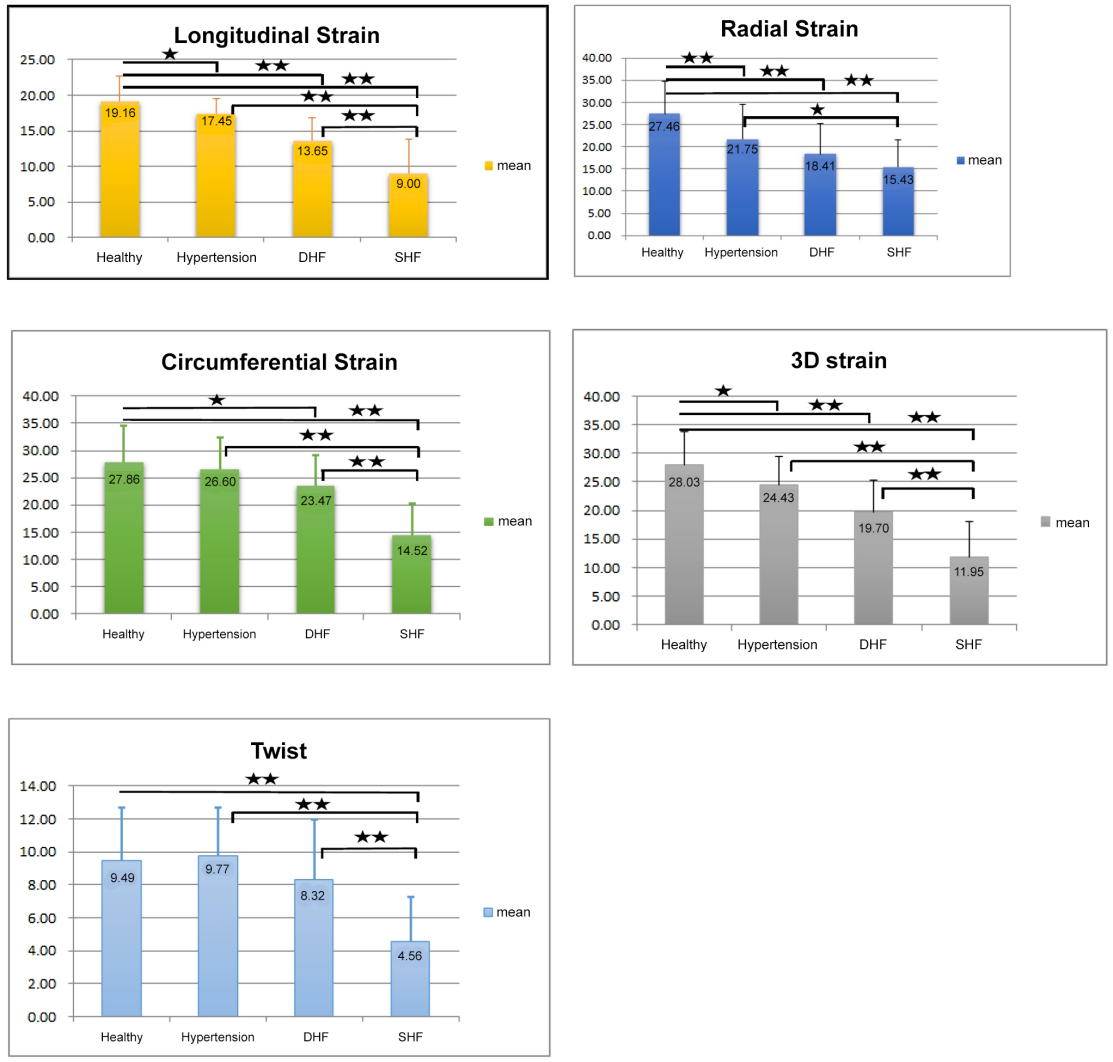
ANOVA *post hoc* analysis revealed significant differences between the mean value of each disease category (★★*p*<0.001, ★*p*<0.05). The original strain parameters in 3D LS and 3D CS were negative and were expressed as the “absolute values” in order to obtain uniform directions with other deformation and twist components.

The baseline conventional echocardiographic parameters are listed in Table 2. Compared to the other groups, the SHF group had a significantly larger LA volume (2D), LVEDV (3D), LVESV (3D), LV mass (with or without index), and PCWP (all *p*<0.001). The SHF group also had significantly lower LVEF (3D) (34.6±7.8, *p*<0.001), IVRT (76.2±14.7, *p*<0.001), DT (191.7±59.3, *p* = 0.0024), and mitral annulus velocity E′ (5.3±2.2, *p*<0.001) values compared to the other disease categories, whereas subjects in the HTN group had the lowest mitral E/A ratio (0.97±0.38). Compared to the control group, the HTN and DHF groups both had a higher ventricular septal wall and relative wall thickness, as well as a lower mitral annulus E′ velocity (all *p*<0.05). In addition, the DHF group demonstrated a higher LV mass (with and without index), as well as a larger myocardial 3D volume and PCWP (E/E′), when compared to the control group (all *p*<0.05). Comparisons between LV global 3D strain and strains from various directions, as well as twist across the four categories are shown in Fig. 2. There were significant differences between the mean values of these four groups for global 3D strain values (28.03, 24.43, 19.70, and 11.95%), LS (−19.16, −17.45, −13.65, and −9%), and RS (27.46, 21.75, 18.41, and 15.43%, all *p*<0.001, respectively). However, the differences were less obvious in the CS (−27.86, −26.6, −23.47, and −14.52%) and twist (9.49°, 9.77°, 8.32°and 4.56°, respectively) between the control and DHF groups (post hoc comparison: *p* = NS; *p* value to reach significance after Bonferroni correction was 0.05/4 = 0.0125). Finally, the differences between the 3D-based myocardial deformation and cardiac twist measurements between the four groups did not change significantly after matching for age, gender, BMI and heart rate variables (S1–S3 Tables in S1 File).

**Table 2:** Baseline echocardiography parameters or diastolic indices.

Clinical Variables	Control (n = 58)	HTN (n = 51)	DHF (n = 61)	SHF (n = 30)	*p* value
LA volume (2D), ml	28.6±8.5	33.5±13.7	40.1±14.3*	46.5±18.7* †	<0.001
IVS, mm	8.2±2.0	9.6±2.5*	10.3±1.9*	9.3±1.2*	<0.001
RWT, mm	0.37±0.08	0.43±0.1* ^§^	0.45±0.08* ^§^	0.35±0.05	<0.001
LVEDV (3D), ml	95.2±23.3	95.8±25.8	110.1±42	173±65.5* † ‡	<0.001
LVESV (3D), ml	44.9±19.3	40.1±18.4	47.6±24.3	112.6±41* † ‡	<0.001
LVEF (3D), %	59.4±12.5	58.7±12.2	57.8±6.9	34.6±7.8* † ‡	<0.001
LV mass^§^, gm	134.8±36.6	170.2±42.7*	181.9±46.5*	214.5±82.3* † ‡	<0.001
LV mass index^§^, gm/m^2^	75.8±19.5	92.6±29*	99.3±30.3*	119.2±44* † ‡	<0.001
Mitral E/A	1.22±0.5	0.97±0.38*	1.06±0.62	1.33±1.02	0.0078
IVRT, ms	83.2±11.8	88.5±17	90.9±14.9*	76.2±14.7†	<0.001
DT, ms	202.8±44	221.7±57.2	228±60.1	191.7±59.3†	0.0024
Mitral annulus velocity S′, cm/s	8.8±3.2	7.7±1.9	7.1±2.4*	5.9±2.0*	<0.001
Mitral annulus velocity E′, cm/s	9.9±2.0	8.4±2.4*	6.2±1.8*	5.3±2.2* †	<0.001
PWCP (estimated by E/E′)	6.9±2	8.6±2.9	13.6±4.7* †	16.9±8.5* † ‡	<0.001

*p<0.05 vs. control group;

† *p*<0.05 vs. HTN group;

‡ *p*<0.05 vs. DHF group; and ^§^ *p*<0.05 vs. SHF group.

Abbreviations: LA: left atrium, 2D: 2-dimensional, IVS: inter-ventricular septum, RWT: relative wall thickness, LVEDV: left ventricular end diastolic volume, 3D: 3-dimensional, LVESV: left ventricular end systolic volume, LVEF: left ventricular ejection fraction, LV: left ventricular, IVRT: isovolumic relaxation time, DT: deceleration time, PWCP: pulmonary capillary wedge pressure.

### Associations between 3D-derived myocardial deformation, cardiac twist, and baseline variables, brain natriuretic peptide or conventional echocardiography parameters

Using linear regression analysis, we examined the associations between global 3D and other strain components, with clinical variables (Table 3) and conventional echo parameters in the 200 subjects (Table 4). Increasing age, higher BNP levels (in log), and poorer renal function (in terms of eGFR) were all associated with a reduction in all strain parameters and cardiac twist (all *p*<0.05). Additionally, subjects with a history of CAD were more likely to have reduced deformation parameters in all directions, as well as decreased cardiac twist (all *p*<0.05). The association between all 3D-derived strain components and serum BNP levels was highly correlated throughout the entire cohort (*r* = 0.61, −0.25, 0.41, −0.46, and −0.32 for LS, RS, CS, 3D, and twist, respectively. All *p*<0.001; data not shown in tables). A clinical history of HTN and a higher heart rate were related to a reduced LS and global 3D strain (both *p*<0.05). Increasing LV wall thickness was also related to reduced myocardial strain and cardiac twist (both *p*<0.05). A reduction in mitral annular diastolic relaxation velocity (E′) and an elevated LV filling pressure were both associated with reduced strain from all directions (all *p*<0.05).

**Table 3:** The associations between all 3D strain, cardiac twist, and baseline clinical variables.

	Longitudinal S, %	Radial S, %	Circumferential S, %	Twist, degree	3D S, %
Variables	Coef. (*p* value)	Coef. (*p* value)	Coef. (*p* value)	Coef. (*p* value)	Coef. (*p* value)
Age, years	−0.104 (<0.001)	−0.15 (<0.001)	−0.09 (0.008)	−0.05 (0.004)	−0.16 (<0.001)
Gender, female	0.43 (0.53)	0.16 (0.89)	−0.68 (0.518)	−0.33 (0.506)	−0.10 (0.922)
Body weight, kg	−0.04 (0.136)	−0.03 (0.942)	−0.003 (0.94)	−0.02 (0.354)	−0.02 (0.681)
BMI, kg/m^2^	−0.18 (0.025)	−0.15 (0.268)	−0.12 (0.323)	−0.06 (0.301)	−0.19 (0.124)
SBP, mmHg	−0.04 (0.042)	−0.06 (0.051)	−0.02 (0.454)	−0.04 (0.02)	−0.06 (0.07)
DBP, mmHg	−0.01 (0.652)	−0.05 (0.279)	0.03 (0.497)	−0.02 (0.628)	−0.02 (0.601)
HR, 1/min	−0.08 (0.002)	−0.1 (0.02)	−0.12 (0.002)	−0.01 (0.567)	−0.13 (0.001)
eGFR	0.07 (<0.001)	0.04 (0.02)	0.06 (0.002)	0.03 (0.001)	0.1 (<0.001)
Hs-CRP (log)	−0.55 (0.022)	0.24 (0.624)	−0.70 (0.077)	−0.39 (0.062)	−0.55 (0.17)
BNP (log)	−1.39 (<0.001)	−0.70 (0.028)	−1.54 (<0.001)	−0.75 (<0.001)	−1.82 (<0.001)
HTN	−2.31 (0.001)	−6.27 (<0.001)	−2.88 (0.06)	−0.4 (0.03)	−4.47 (<0.001)
DM	−1.6 (0.042)	−4.9 (<0.001)	−2.55 (0.035)	−1.0 (0.087)	−3.91 (0.001)
Hyperlipidemia	−2.42 (0.002)	−3.5 (0.01)	−1.67 (0.171)	0.05 (0.927)	−2.86 (0.02)
CAD	−4.57 (<0.001)	−5.55 (<0.001)	−4.25 (<0.001)	−1.52 (0.01)	−6.59 (<0.001)

Data are expressed as coefficient (*p* value).

Abbreviations: BMI: body mass index, SBP: systolic blood pressure, DBP: diastolic blood pressure, HR: heart rate, eGFR: estimated glomerular filtration rate, BNP: brain natriuretic peptide, HTN: hypertension. DM: diabetes, CAD: coronary artery disease.

The strain parameters were expressed and calculated as the “absolute values” in 3D longitudinal strain and 3D circumferential strain.

**Table 4:** The associations between all 3D strain, cardiac twist, and baseline echocardiography parameters or diastolic indices.

	Longitudinal S, %	Radial S, %	Circumferential S, %	Twist, degree	3D S, %
Variables	Coef. (*p* value)	Coef. (*p* value)	Coef. (*p* value)	Coef. (*p* value)	Coef. (*p* value)
LA volume (2D), ml	−0.12 (<0.001)	−0.15 (<0.001)	−0.1 (0.002)	−0.07 (<0.001)	−0.16 (<0.001)
IVS, mm	−0.36 (0.003)	0.63 (0.014)	0.48 (0.034)	0.12 (0.02)	0.25 (0.3)
RWT	−4.21 (<0.001)	1.65 (0.005)	1.75 (0.001)	0.88 (0.001)	1.32 (0.014)
LVEF (3D), %	0.20 (<0.001)	0.13 (0.003)	0.38 (<0.001)	0.13 (<0.001)	0.29 (<0.001)
LV mass index, g/m^2^ §	−0.04 (<0.001)	−0.01 (0.625)	0.04 (0.018)	−0.03 (<0.001)	−0.04 (0.004)
Myocardial 3D volume index, ml/m^2^	−0.05 (<0.001)	−0.01 (0.533)	−0.06 (0.001)	−0.05 (<0.001)	−0.06 (0.001)
Mitral E/A	−1.38 (0.008)	−0.85 (0.36)	−2.31 (0.004)	−0.41 (0.307)	−1.69 (0.039)
IVRT, ms	0.03 (0.229)	0.01 (0.714)	0.06 (0.065)	0.02 (0.359)	0.06 (0.101)
DT, ms	0.01 (0.033)	0.004 (0.672)	0.03 (<0.001)	0.005 (0.242)	0.02 (0.07)
Mitral annulus velocity S′, cm/s	0.43 (0.001)	0.51 (0.019)	0.66 (0.001)	0.14 (0.128)	0.77 (<0.001)
Mitral annulus velocity E′, cm/s	0.95 (<0.001)	1.24 (<0.001)	0.86 (<0.001)	0.41 (<0.001)	1.44 (<0.001)
PWCP (estimated E/E′)	−0.47 (<0.001)	−0.58 (<0.001)	−0.58 (<0.001)	−0.19 (<0.001)	−0.73 (<0.001)

§ M-mode method.

Data are expressed as coefficient (*p* value).

Abbreviations as in Table 2.

The strain parameters were expressed and calculated as the “absolute values” in 3D longitudinal strain and 3D circumferential strain.

### The clinical prognostic value of 3D-based strain parameters and cardiac twist

For all participants with registered final follow-up data, only the 1^st^ cohort entered the follow-up period with sufficient observation intervals (with 98% survival data available: One control and one SHF subject was lost during follow-up). The predictions of cardiac death or HF hospitalization using different methodologies of 3D echocardiography were determined (Table 5). A total of 29 such events occurred during a mean follow-up of 567.7 days (median: 725.5 days; 25^th^–75^th^ IQR: 197–909 days). In the unadjusted model, all 3D-based measures (3D LS, 3D RS, 3D CS, 3D strain, and 3D twist), conferred a significantly increased HR for clinical events (all *p*<0.05). After an adjustment of clinical variables such as age and sex with 3D-defined LVEF, the HR remained independently as 1.16 (95% CI: 1.04–1.29) for 3D LS and 1.07 (95% CI: 1.10–1.15) for 3D RS. Additionally, HRs were estimated to be 1.22 (95% CI: 1.13–1.32) for 3D LS, 1.10 (95% CI: 1.02–1.17) for 3D RS, 1.13 (95% CI: 1.07–1.20) for 3D CS, 1.16 (95% CI: 1.08–1.23) for global 3D strain, and 1.15 (95% CI: 1.02–1.29) for 3D twist when LV mass (M-mode) was adjusted together with clinical age and sex (all *p*<0.05). Similar results were observed if the 3D-based myocardial volume as well as age and sex were adjusted (all *p*<0.05).

**Table 5:** The prognostic value of various deformation components derived from 3D software.

Deformation or Twist	Longitudinal S, %	Radial S, %	Circumferential S, %	3D S, %	Twist
Hazard ratio (95% CI)					
Un-adjusted model	1.23 (1.15 to 1.31)*	1.11 (1.05 to 1.18)*	1.16 (1.1 to 1.23)*	1.18 (1.11 to 1.25)*	1.21 (1.09 to 1.35)*
Multi-variable adjusted models					
Model 1	1.22 (1.13 to 1.31)*	1.11 (1.04 to 1.18)*	1.14 (1.08 to 1.21)*	1.17 (1.1 to 1.24)*	1.19 (1.07 to 1.32)*
Model 2	1.21 (1.13 to 1.30)*	1.10 (1.04 to 1.18)*	1.14 (1.08 to 1.2)*	1.16 (1.09 to 1.23)*	1.18 (1.05 to1.31)*
Model 2 + LVEF (3D)	1.16 (1.04 to 1.29)*	1.07 (1.0 to 1.15)*	1.08 (0.98 to 1.18)	1.11 (1.02 to 1.20)*	1.0 (0.88 to 1.16)
Model 2 + LV Mass (M-mode)	1.22 (1.13 to 1.32)*	1.10 (1.02 to 1.17)*	1.13 (1.07 to 1.20)*	1.16 (1.08 to 1.23)*	1.15 (1.02 to1.29)*

*p<0.05.

§ *p*≥0.05 and *p*<0.1.

Abbreviations: 3D: 3-dimensional, S: strain.

Model 1: adjusted for age; Model 2: adjusted for age, and sex.

The strain parameters were expressed and calculated as the “absolute values” in 3D-derived longitudinal and circumferential strains.

**Figure 3:**
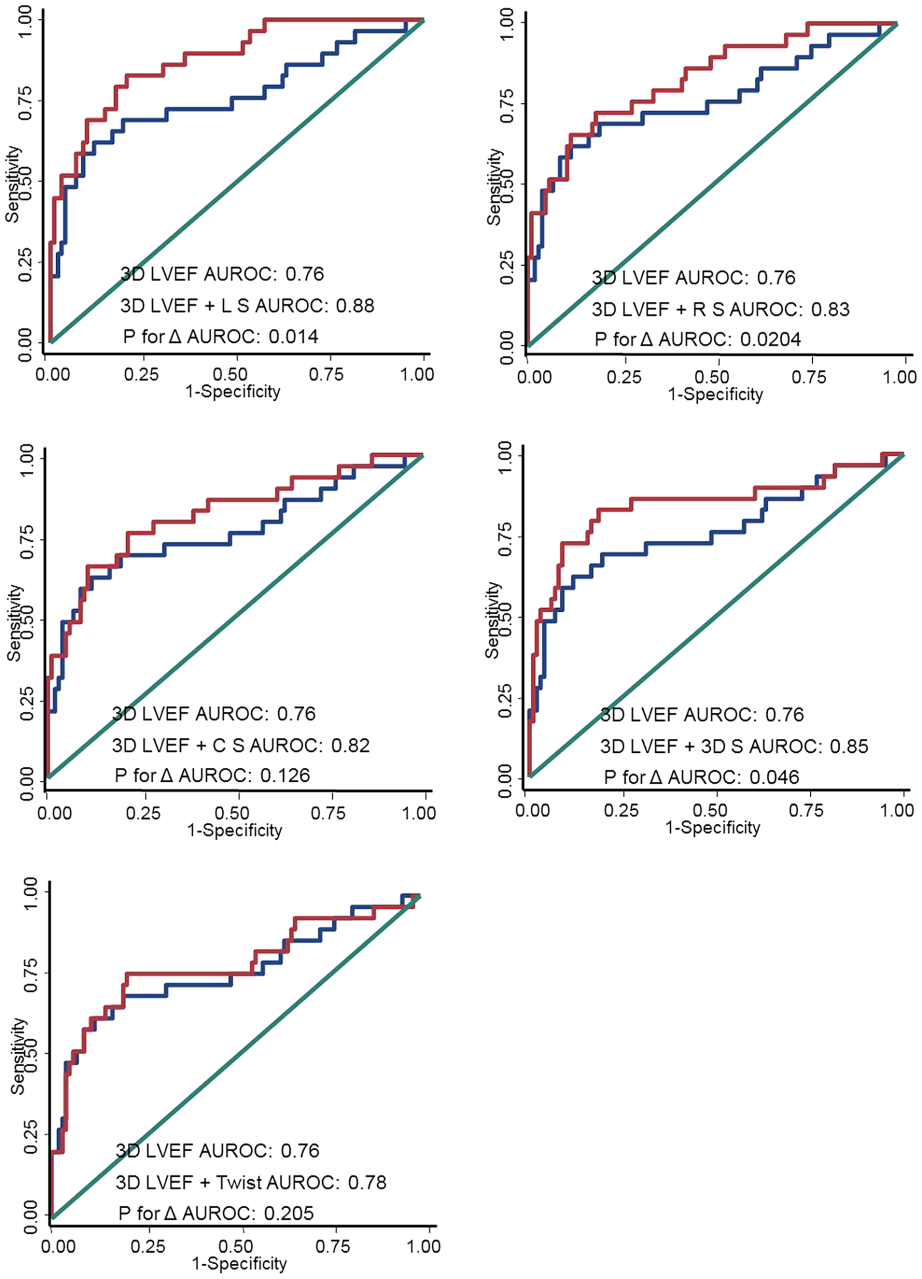
The corresponding AUROC generated in predicting clinical outcomes when superimposed on 3D LVEF by 3D-based LS, RS, CS, 3D strain, and twist, respectively. 3D LS, 3D RS, and 3D strain yielded significant incremental values in the clinical outcome prediction model beyond 3D LVEF (all *p* for delta AUROC: <0.05).

The cut-off values of each parameter for predicting clinical outcomes were analyzed by using the ROC method (summarized in Table 6). The incrementally predictive values for all individual 3D-based deformations superimposed on LVEF (3D-based) were also analyzed (Fig. 3), with the significance of AUROC changes (delta) expressed. When 3D LS, 3D RS, or global 3D strain were superimposed on the original 3D LVEF, there were significant incremental values in the outcome prediction models (all *p* for delta AUROC <0.05).

**Table 6:** The cut-off values of each strain parameter or twist for clinical outcome prediction.

Deformation or Twist	Longitudinal S, %	Radial S, %	Circumferential S, %	3D S, %	Twist
Cut point	−13.31	18.58	−19.45	18.28	9.01
Sensitivity	79.31%	89.66%	68.97%	86.21%	93.10%
Specificity	82.52%	58.25%	84.47%	75.73%	55.34%
Correctly Classified	81.82%	65.15%	81.06%	78.03%	63.64%

Abbreviations: 3D: 3-dimensional, S: strain.

**Figure 4:**
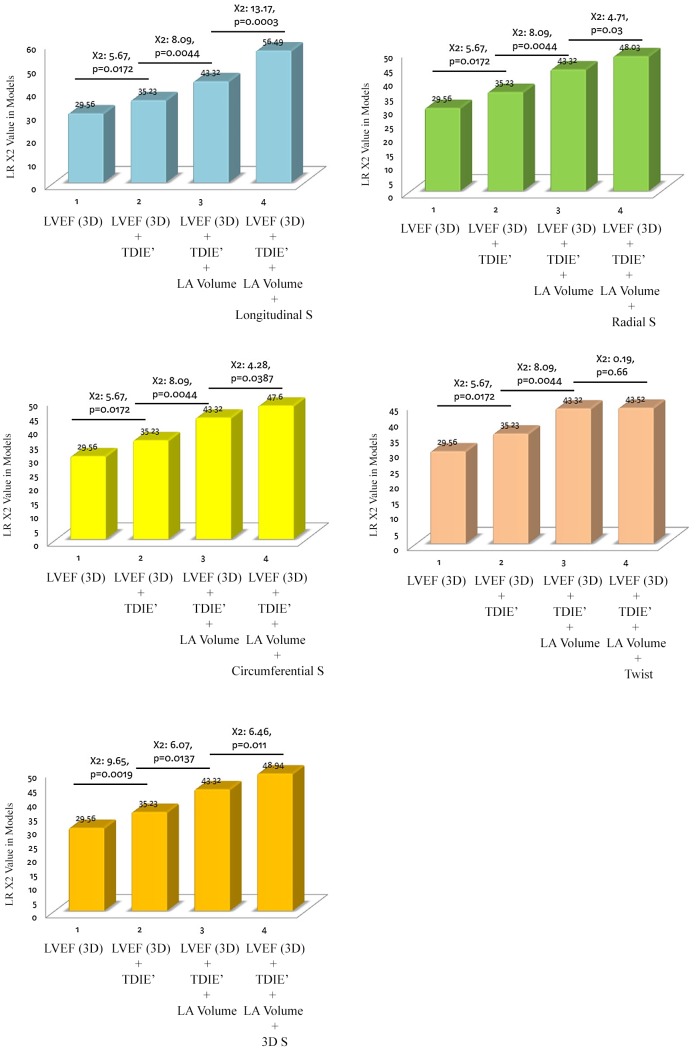
The incremental *X* ^2^ value for 3D-based LS was 13.17 (*p* = 0.0003), which was higher than that of the 3D strain (6.46, *p* = 0.011), 3D RS (4.71, *p* = 0.03), and 3D CS (4.28, *p* = 0.0387), after combining the 3D LVEF, 2D TDI E′, and 2D LA volumes.

The likelihood *X* ^2^ test also showed that 3D LS, 3D RS, 3D CS, and 3D strain added a significant incremental value (*X^2^* = 13.17, *p* = 0.0003 for 3D LS, *X^2^* = 6.46, *p* = 0.011 for global 3D strain, *X^2^* = 4.71, *p* = 0.03 for 3D, and *X^2^* = 4.28, *p* = 0.0387 for 3D CS) on clinical outcome predictions when superimposed on 3D LVEF, 2D TDI E′, and 2D LA volumes, respectively (Fig. 4).

## Discussion

In this study we have demonstrated that 3D echocardiography-derived myocardial deformation parameters (particularly global 3D strain, and 3D-derived longitudinal and radial strain indices) can aid in characterizing the extent of myocardial dysfunction in subjects with HTN or HF (both systolic and diastolic). In line with previous reports, we observed that graded longitudinal functional decline may occur in subjects with HTN or DHF, which is in part compensated by relatively well preserved short-axis function or twist [16]. However, an ultimate mechanical failure of all myocardial deformation components, including LV twist, may occur upon reaching SHF status [27]. Furthermore, we demonstrated that these 3D-based myocardial deformation parameters may improve risk stratification when superimposed on conventional echocardiography measures, including LV ejection fraction or LV mass.

Although the numbers in this study were small, it appeared that the subjects in the HTN group were at a stage of more impaired diastolic relaxation, denoted by an inverted E/A ratio. Notably, HF with preserved LVEF, namely DHF, has comprised nearly half of all patients with HF in recent years [28]. The lack of an effective treatment for this patient population may be partly due to the limited knowledge of altered myocardial mechanics. Recent advances in echocardiography, especially in myocardial deformation imaging derived from 2D speckle tracking, has substantially improved our understanding of various systolic myocardial mechanics [29]. However, thus far there is limited data regarding how these individual systolic mechanical indices may correlate with clinical variables.

Compared to 2D-derived myocardial deformation, 3D-based strain measurement is a novel and newly developed technique that can help to assess cardiac mechanics more rapidly and in a more comprehensively detailed and reproducible manner [18], [30]. The method shares some features with 2D-STI but is capable of the simultaneous analysis of both global and segmental myocardial functions, and it thus provides better spatial complexity with reasonable resolution [18], [30]. In addition, global 3D strain is regarded as a true 3D-based myocardial deformation measure, where dedicated real tracking of two adjacent points in the myocardium is performed. This measure also provides a global evaluation of 3D-based myocardial contractile mechanics that is not limited to the directions or planes of cardiac motions. It may therefore provide a more comprehensive understanding of myocardial mechanics.

In previous studies, LVEF obtained by transthoracic 2D echocardiography has emerged as the most important clinical variable in determining the extent of systolic dysfunction for guiding therapeutic decisions [31]. However, we suggest that 2D LVEF may not be sensitive enough to identify the early stages of myocardial dysfunction. In addition, it may be significantly influenced by ventricular loading. Furthermore, diastolic functional assessments by TDI techniques are limited by their angle-dependency, their inability to fully assess global cardiac function, and the wide overlap of pure diastolic dysfunction and overt diastolic HF [32]. In this study, we have demonstrated that a simple global 3D strain measurement may be sufficient for HF risk stratification in daily clinical practice. Notably, when 3D strain was added to conventional echo measures, including diastolic function and LA volume, a superior prognostic value was still observed.

### Study Limitations

There are several limitations to this cohort study. Our data and conclusions may need to be interpreted with caution, in part because of the study's retrospective nature and the relatively small numbers used in both the disease and control groups. Important risk factors (e.g., smoking, diabetes, renal dysfunction, drug therapy, etc.), which were not considered due to the small number of clinical events, may also limit the study's validity. Furthermore, renal function, and other medical histories of the DHF group did not match the other study groups, even after matching for age, gender, BMI and heart rate. This may potentially make the comparisons between the study and control groups invalid. Therefore, we suggest that additional larger, more rigorous, and more comprehensive prospective studies be conducted in the future.

## Conclusions

This clinical study aimed to examine the relationship between LV function and mechanics in subjects with a variety of clinical conditions, by using novel 3D-based myocardial tracking techniques. We proved this technique to be clinically feasible and that it can aid in characterizing the extent of myocardial dysfunction in various pathophysiological states. We also successfully demonstrated that 3D strain based measures were superior to LVEF in predicting clinical outcomes, including HF events and life expectancy.

## Supporting Information

S1 Fig A flow chart of the current study, including the initial total number of participants (n = 214). Fourteen were excluded due to poor image quality, unavailable baseline information (n = 1), or two loss in the follow-up. The final total baseline study cohort number was 200. During a mean 567.7 days of follow-up period, there were 131 subjects who had follow-up data available.
(TIF)

S2 Fig An illustration of automatic strain analysis. Strain curves for the apical four-chamber view shows A: longitudinal strain, B: radial strain, C: circumferential strain, and D: 3D strain, respectively, in the healthy subjects.
(JPG)

S3 Fig An illustration of automatic strain analysis. Strain curves for the apical four-chamber view shows A: longitudinal strain, B: radial strain, C: circumferential strain, and D: 3D strain, respectively, in the heart failure subjects.
(JPG)

S4 Fig Automatic twist analysis of the left ventricle. Twist curves of the apical four-chamber view shows A: healthy subjects, and B: heart failure subjects.
(JPG)

S1 File Contains the following files: **S1 Table.** Baseline demographic characteristics of the study subjects after matching. *p<0.05 vs. Control group; ^†^ *p*<0.05 vs. HTN group; ^‡^ *p*<0.05 vs. DHF group; and ^§^ *p*<0.05 vs. SHF group. Abbreviations as in Table 1. **S2 Table.** Baseline key echocardiography parameters or diastolic indices after matching. *p<0.05 vs. Control group; ^†^ *p*<0.05 vs. HTN group; ^‡^ *p*<0.05 vs. DHF group; and ^§^ *p*<0.05 vs. SHF group. Abbreviations as in Table 2. **S3 Table.** Comparisons of 3D-based deformations and cardiac twist after matching. *p<0.05 vs. control group; ^†^ *p*<0.05 vs. HTN group; ^‡^ *p*<0.05 vs. DHF group; and ^§^ *p*<0.05 vs. SHF group.
(DOCX)
