# Correction: Cardiac Mechanics and Ventricular Twist by Three-Dimensional Strain Analysis in Relation to B-Type Natriuretic Peptide as a Clinical Prognosticator for Heart Failure Patients

**DOI:** 10.1371/journal.pone.0123049

**Published:** 2015-04-01

**Authors:** The PLOS ONE Staff

A second affiliation for the second author is not indicated. Yau-Huei Lai is also affiliated with Mackay Junior College of Medicine, Nursing, and Management, New Taipei City, Taiwan.

The following information is missing from the Funding section: This work was partially funded by grants from National Science Council (NSC-101-2314-B-195-020, NSC 103-2314-B-010-005-MY3, 103-2314-B-195-001-MY3, 101-2314-B-195-020-MY1, MOST 103-2314-B-195-006-MY3), Mackay Memorial Hospital (10271, 10248, 10220, 10253, 10375, 10358, E-102003) and Taiwan Foundation for geriatric emergency and critical care.

## References

[1] Chang S-N, Lai Y-H, Yen C-H, Tsai C-T, Lin J-W, Bulwer BE, et al (2014) Cardiac Mechanics and Ventricular Twist by Three-Dimensional Strain Analysis in Relation to B-Type Natriuretic Peptide as a Clinical Prognosticator for Heart Failure Patients. PLoS ONE 9(12): e115260 doi: 10.1371/journal.pone.0115260 25545637 10.1371/journal.pone.0115260 PMC4278904

