# Supplementary figures and images for: Cardiac Mechanics and Ventricular Twist by Three-Dimensional Strain Analysis in Relation to B-Type Natriuretic Peptide as a Clinical Prognosticator for Heart Failure Patients
Source: PLoS One. 2014 Dec 29;9(12):e115260. doi: 10.1371/journal.pone.0115260 (PMC4278904; doi:10.1371/journal.pone.0115260)

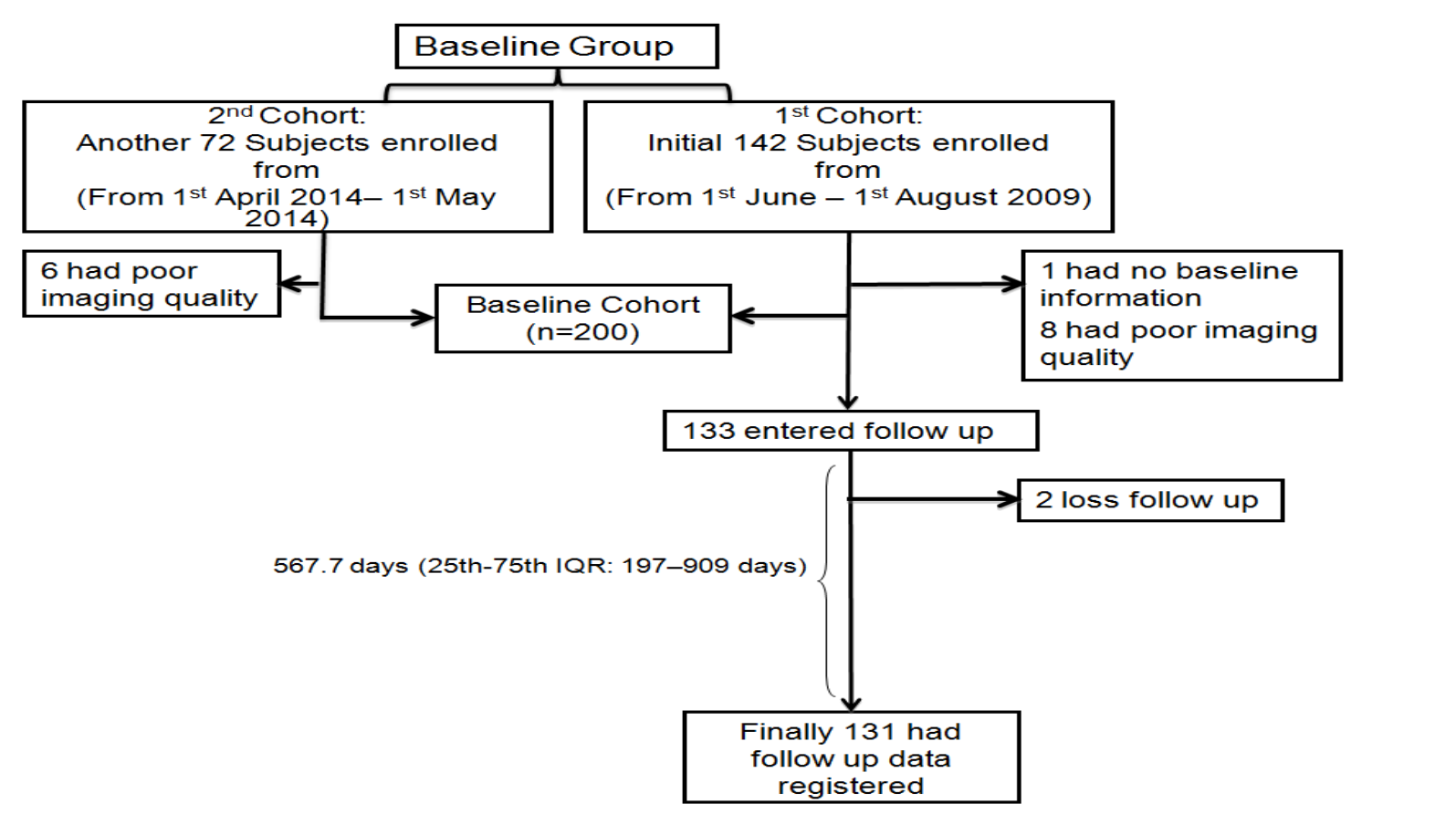

Supplement: S1 Fig — A flow chart of the current study, including the initial total number of participants (n = 214). Fourteen were excluded due to poor image quality, unavailable baseline information (n = 1), or two loss in the follow-up. The final total baseline study cohort number was 200. During a mean 567.7 days of follow-up period, there were 131 subjects who had follow-up data available. (TIF) [file pone.0115260.s001.tif]

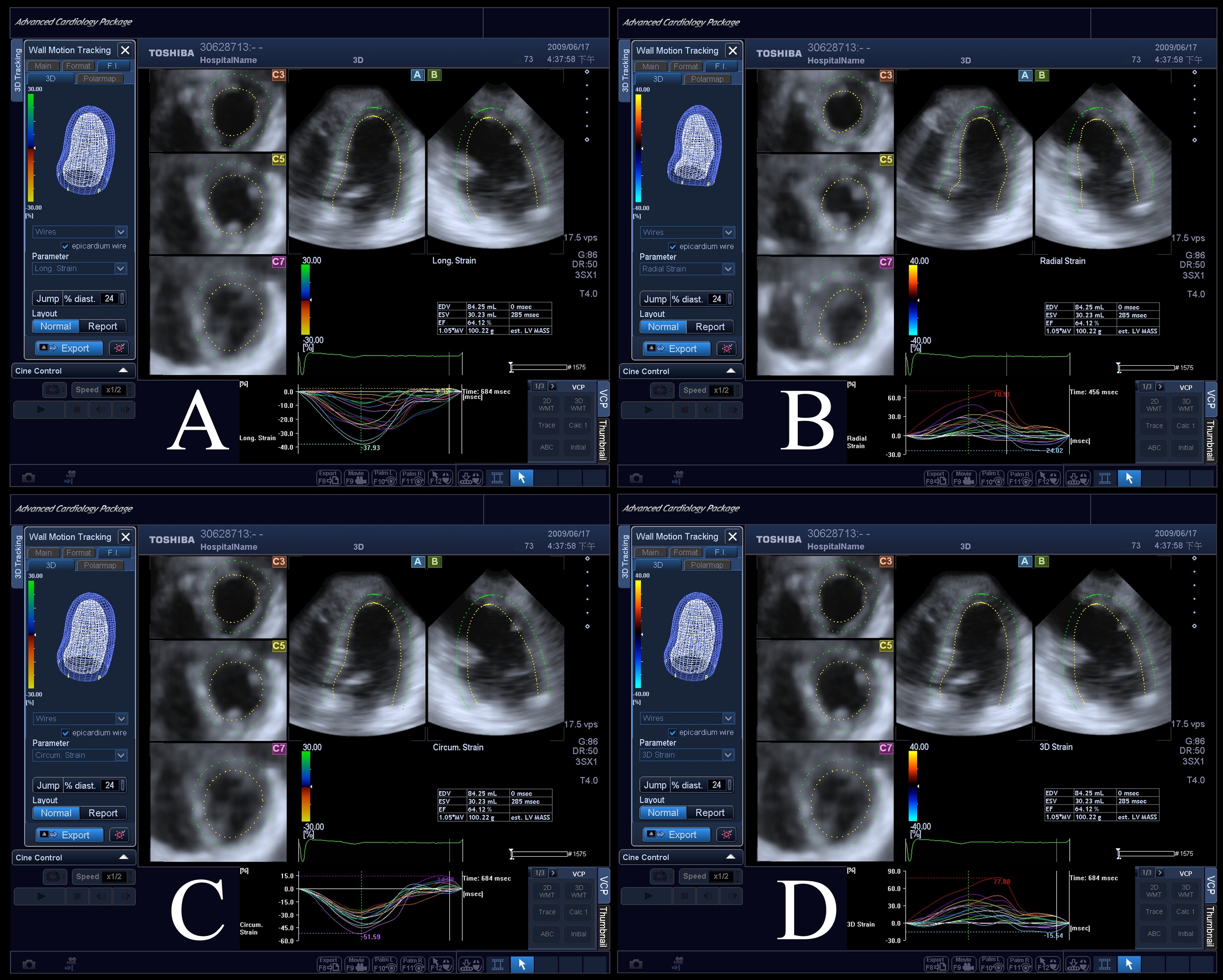

Supplement: S2 Fig — An illustration of automatic strain analysis. Strain curves for the apical four-chamber view shows A: longitudinal strain, B: radial strain, C: circumferential strain, and D: 3D strain, respectively, in the healthy subjects. (JPG) [file pone.0115260.s002.jpg]

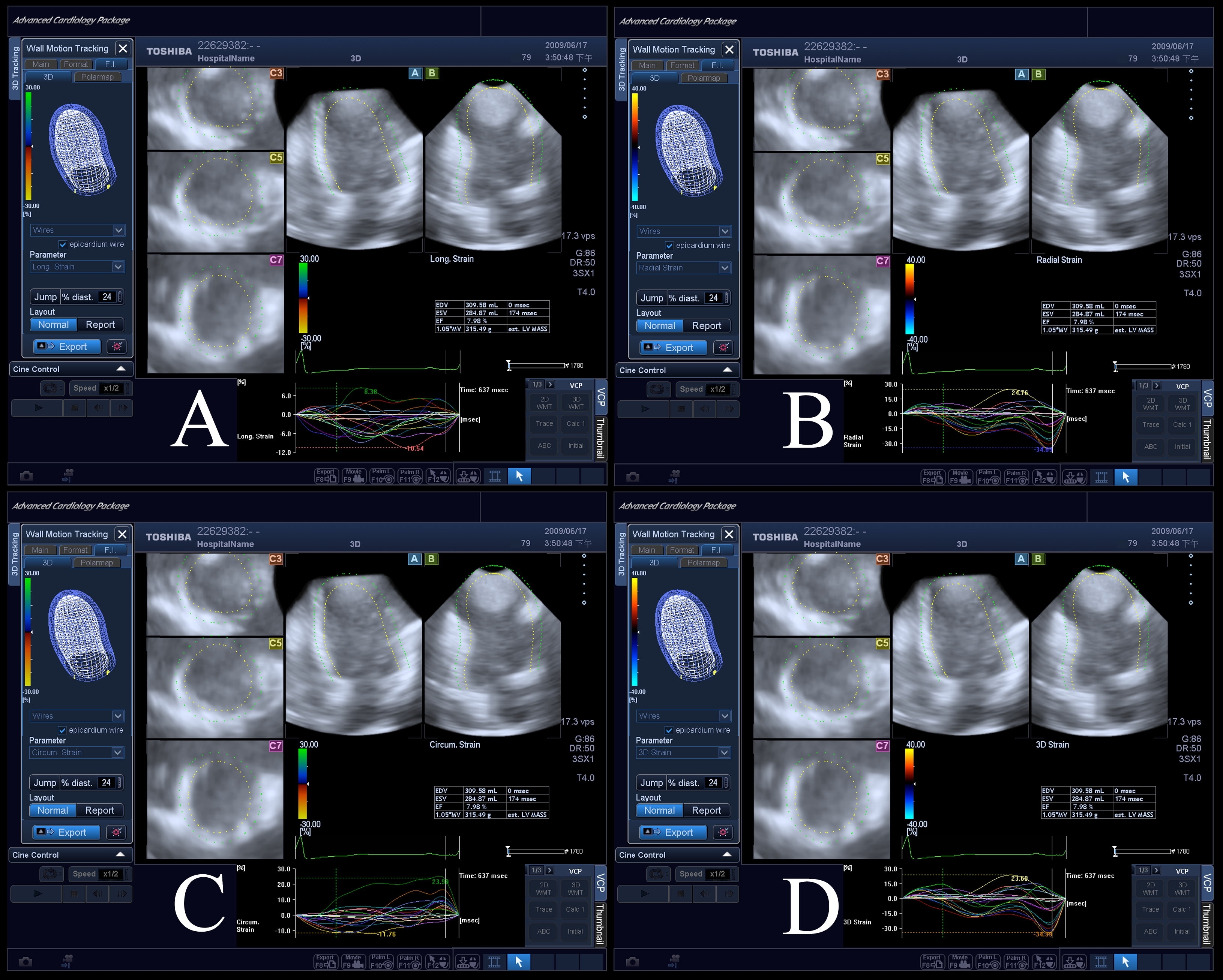

Supplement: S3 Fig — An illustration of automatic strain analysis. Strain curves for the apical four-chamber view shows A: longitudinal strain, B: radial strain, C: circumferential strain, and D: 3D strain, respectively, in the heart failure subjects. (JPG) [file pone.0115260.s003.jpg]

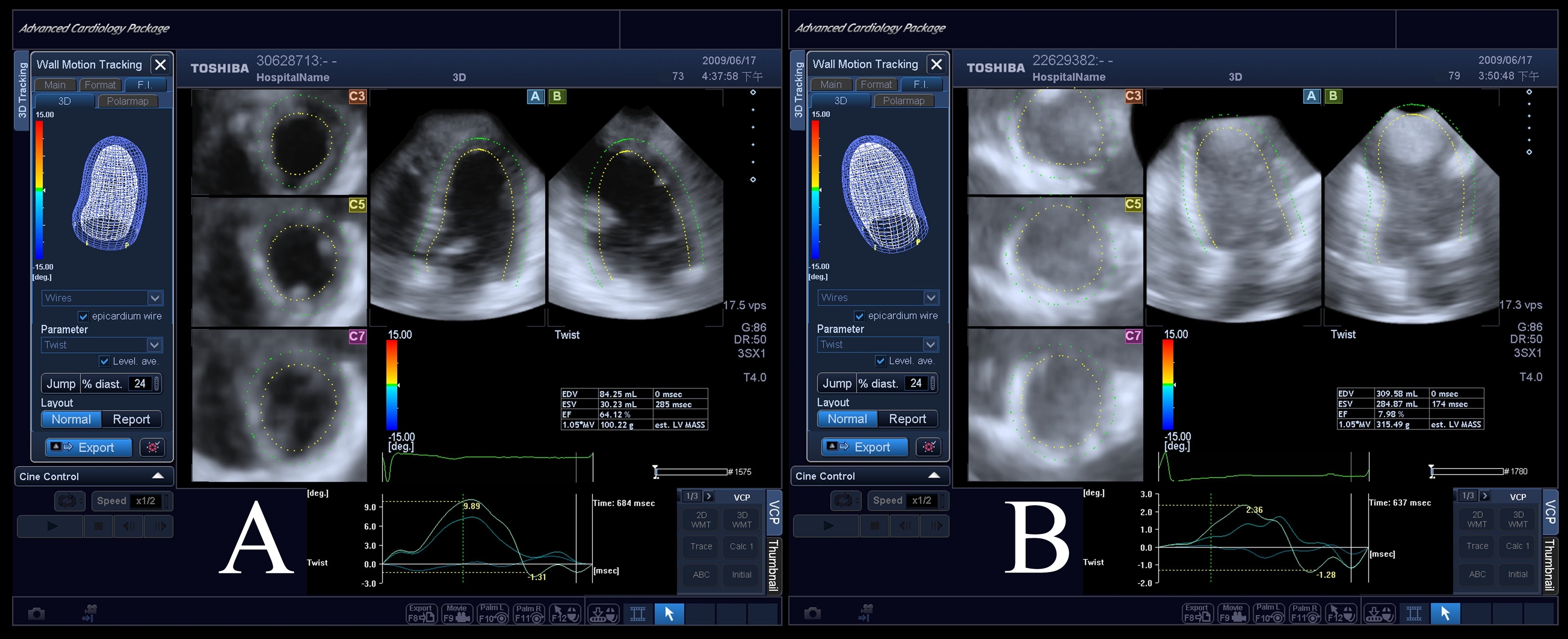

Supplement: S4 Fig — Automatic twist analysis of the left ventricle. Twist curves of the apical four-chamber view shows A: healthy subjects, and B: heart failure subjects. (JPG) [file pone.0115260.s004.jpg]
