# Supplementary material for: Cardiac Mechanics and Ventricular Twist by Three-Dimensional Strain Analysis in Relation to B-Type Natriuretic Peptide as a Clinical Prognosticator for Heart Failure Patients
Source: PLoS One. 2014 Dec 29;9(12):e115260. doi: 10.1371/journal.pone.0115260 (PMC4278904; doi:10.1371/journal.pone.0115260)
Supplement: S1 File — Contains the following files: S1 Table. Baseline demographic characteristics of the study subjects after matching. *p<0.05 vs. Control group; † p<0.05 vs. HTN group; ‡ p<0.05 vs. DHF group; and § p<0.05 vs. SHF group. Abbreviations as in Table 1. S2 Table. Baseline key echocardiography parameters or diastolic indices after matching. *p<0.05 vs. Control group; † p<0.05 vs. HTN group; ‡ p<0.05 vs. DHF group; and § p<0.05 vs. SHF group. Abbreviations as in Table 2. S3 Table. Comparisons of 3D-based deformations and cardiac twist after matching. *p<0.05 vs. control group; † p<0.05 vs. HTN group; ‡ p<0.05 vs. DHF group; and § p<0.05 vs. SHF group. (DOCX) [file pone.0115260.s005.docx]

**Supplemental Tables**

**S1 Table. Baseline demographic characteristics of the study subjects after matching**

| **Clinical Variables** | **Control (n = 27)** | **HTN (n = 38)** | **DHF (n = 36)** | **SHF (n = 21)** | **ANOVA *p* value** |
| --- | --- | --- | --- | --- | --- |
| Age, years | 62.2 ± 8.3 | 62.4 ± 9.3 | 62.4 ± 12.3 | 64.1 ± 16.3 | 0.934 |
| Gender, male | 11 (41%) | 22 (57.9%) | 12 (33.3%) | 11 (52.4%) | 0.162 |
| BMI, kg/m^2^ | 24.5 ± 2.7 | 25.3 ± 3.5 | 26.5 ± 2.5 | 24.8 ± 2.6 | 0.07 |
| SBP, mmHg | 130.8 ± 20.4 | 138.3 ± 22.9 | 138.4 ± 22.7 | 142.1 ± 27.1 | 0.36 |
| DBP, mmHg | 76.3 ± 11.4 | 80.2 ± 13 | 79.6 ± 13.1 | 77.6 ± 12.7 | 0.613 |
| HR, 1/min | 74.3 ± 7.6 | 81.3 ± 11.9 | 78.2 ± 13.4 | 78.8 ± 13.6 | 0.147 |
| eGFR, ml/min/1.73 m^2^ | 83.7 ± 20.3 | 83.6 ± 25.9 | 80.7 ± 30.6^＊^ | 62.5 ± 26.7^＊†^ | 0.0252 |
| BNP (log) | 2.4 ± 1.4 | 2.4 ± 1.2 | 3.9 ± 1.7^＊†^ | 5.2 ± 1.4^＊†‡^ | <0.001 |
| HTN, % | — | 38 (100%) | 24 (67%) | 14 (67%) | <0.001 |
| DM, % | — | 16 (31%) | 12 (33.3%) | 6 (29%) | 0.002 |
| Hyperlipidemia, % | — | 16 (42.1%) | 12 (33.3%) | 8 (38.1%) | 0.002 |
| CAD, % | — | 7 (18.4%) | 14 (38.9%) | 10 (47.6%) | <0.001 |

^＊^*p* < 0.05 vs. Control group; ^†^*p* < 0.05 vs. HTN group; ^‡^*p* < 0.05 vs. DHF group; and ^§^*p* < 0.05 vs. SHF group.

Abbreviations as in Table 1.

**S2 Table. Baseline key echocardiography parameters or diastolic indices after matching**

| **Clinical Variables** | **Control (n = 27)** | **HTN (n = 38)** | **DHF (n = 36)** | **SHF (n = 21)** | **ANOVA *p* value** |
| --- | --- | --- | --- | --- | --- |
| LA volume (2D), ml | 30.2 ± 8.4 | 34.4 ± 15.3 | 40 ± 14^＊^ | 44.4 ± 12.5^＊†^ | <0.001 |
| LVEF (3D), % | 58.5 ± 12.3 | 59.9 ± 12.8 | 57.2 ± 6.3 | 34.9 ± 6.4^＊†‡^ | <0.001 |
| LV mass index^§^, gm/m^2^ | 76.5 ± 21.9 | 90.3 ± 26^＊^ | 99.2 ± 36.1^＊^ | 121.4 ± 34.7^＊†‡^ | <0.001 |
| Mitral E/A | 1.03 ± 0.5 | 0.9 ± 0.38 | 0.98 ± 0.42 | 1.37 ± 0.81^†‡^ | 0.0129 |
| IVRT, ms | 85.3 ± 12.5 | 89.7 ± 15.5 | 87.8 ± 14 | 74 ± 11^＊†‡^ | <0.001 |
| DT, ms | 206.5 ± 46.3 | 228.1 ± 61.6 | 226.3 ± 66.7 | 187 ± 50.5^†^ | 0.046 |
| Mitral annulus velocity S', cm/s | 8.9 ± 4.8 | 7.5 ± 1.8 | 7.3 ± 2.5^＊^ | 5.9 ± 1.7^＊^ | 0.0061 |
| Mitral annulus velocity E', cm/s | 8.8 ± 1.6 | 7.9 ± 2.1 | 6.4 ± 2^＊†^ | 6.17 ± 2.3^＊†^ | <0.001 |
| PWCP (estimated by E/E’) | 7.3 ± 1.7 | 9 ± 2.5 | 12.5 ± 5.3^＊†^ | 16.5 ± 7.4^＊†‡^ | <0.001 |

^＊^*p* < 0.05 vs. Control group; ^†^*p* < 0.05 vs. HTN group; ^‡^*p* < 0.05 vs. DHF group; and ^§^*p* < 0.05 vs. SHF group.

Abbreviations as in Table 2.

**S3 Table. Comparisons of 3D-based deformations and cardiac twist after matching**

| **Clinical Variables** | **Control (n = 27)** | **HTN (n = 38)** | **DHF (n = 36)** | **SHF (n = 21)** | **ANOVA *p* value** |
| --- | --- | --- | --- | --- | --- |
| Longitudinal Strain, % | 19.4 ± 3.4 | 17.2 ± 1.8^＊^ | 13.6 ± 2.9^＊†^ | 10.2 ± 4.8^＊†‡^ | <0.001 |
| Radial Strain, % | 28.5 ± 8.6 | 21 ± 7.3^＊^ | 18.4 ± 6.9^＊^ | 17.5 ± 6.5^＊^ | <0.001 |
| Circumferential Strain, % | 29.2 ± 7.3 | 26.5 ± 6 | 23.5 ± 5.7^＊^ | 16.1 ± 5.7^＊†‡^ | <0.001 |
| 3D Strain % | 28.5 ± 5.4 | 24 ± 4.9^＊^ | 19 ± 5.4^＊†^ | 14.3 ± 6^＊†‡^ | <0.001 |
| Twist, ° | 8.7 ± 3 | 9.8 ± 2.5 | 8.4 ± 3.7 | 5.9 ± 2.8^＊†‡^ | <0.001 |

^＊^*p* < 0.05 vs. control group; ^†^*p* < 0.05 vs. HTN group; ^‡^*p* < 0.05 vs. DHF group; and ^§^*p* < 0.05 vs. SHF group.
